# Retrospective analysis of the ^18^F-FDG PET/CT cutoff value for metabolic parameters was performed as a prediction model to evaluate risk factors for endometrial cancer

**DOI:** 10.1186/s13014-023-02382-6

**Published:** 2023-12-04

**Authors:** Ye Yang, Yu-Qin Pan, Min Wang, Song Gu, Wei Bao

**Affiliations:** 1 Obstetrics and Gynecology Department, Shanghai General Hospital, Shanghai Jiao Tong University School of Medicine, 85 Wujin Road, Hongkou, Shanghai, 200080, 8615921055641 P.R. China; 2 Surgical Department, Shanghai General Hospital, Shanghai Jiao Tong University School of Medicine, Shanghai, P.R. China; 3 General Surgery Department, Shanghai General Hospital, Shanghai Jiao Tong University School of Medicine, Shanghai, P.R. China; 4 Trauma Center, Shanghai General Hospital, Shanghai Jiao Tong University School of Medicine, 650 Xin Songjiang Road, Songjiang, Shanghai, 201620 P.R. China

**Keywords:** Endometrial cancer, ^18^F-FDG PET/CT, Standardized uptake value (SUV), Metabolic Tumor volume (MTV), Total lesion glycolysis (TLG), Deep myometrial invasion (DMI), Endocervical stroma invasion (ESI), Lymph node metastases (LNM)

## Abstract

**Purpose:**

The study retrospectively analyzed the accuracy and predictive ability of preoperative integrated whole-body ^18^F-FDG PET/CT for the assessment of high-risk factors in patients with endometrial carcinoma (EC).

**Materials and methods:**

A total of 205 patients with endometrial cancer who underwent preoperative PET/CT at Shanghai General Hospital from January 2018 to December 2021 were retrospectively evaluated and last follow-up was June 2023. Our study evaluated the ability and optimal cutoff values of three metabolic and volumetric parameters—standardized uptake value (SUV), metabolic tumor volume (MTV) and total lesion glycolysis (TLG)—to predict deep myometrial invasion (DMI), endocervical stroma invasion (ESI) and lymph node metastases (LNM) in endometrial cancer. The accuracy, sensitivity, specificity, positive predictive value (PPV), and negative predictive value (NPV) of PET/CT were used to assess the diagnostic performance for the prediction.

**Results:**

Our study demonstrated a significant relationship between SUVmax (11.29, 17.38, 9.47), SUVmean (5.20, 6.12, 4.49), MTV (38.15, 36.28, 33.79 ml), and TLG (199.30, 225.10, 156.40 g) on PET/CT and histologically confirmed DMI, ESI and LNM in endometrial carcinoma (EC), with sensitivity, specificity, accuracy, PPV, and NPV of 100%/100%/100%, 96.53%/98.89%/87.14%, 97.56%/99.02%/91.22%, 92.42%/92.85%/78.31%, and 100%/100%/100%, respectively. Our study showed a risk model based on optimal cutoff values for MTV and TLG of 19.6 ml/126.3 g, 20.54 ml/84.80 g and 24 ml/49.83 g to preoperatively predict DMI, ESI, and LNM, respectively, in endometrial carcinoma. The 4-year OS (HR) for Stage IA, IB, II, III and IV according to 2009 FIGO was 98.00% (0.22), 95.20% (0.04), 83.90% (0.18), 90.50% (0.09) and 60% (0.51). Accordingly, estimated 4-year DFS (HR) for the stage IA-III was 98% (0.02), 95.20% (0.05), 76.90% (0.27) and 76.30% (0.35), all the patients in stage IV occurred recurrence and progression.

**Conclusion:**

The present study showed patients with MTV > = 19.6 ml of MI and PET- positive LN with MTV cutoff > = 24 ml tended to predict poor OS and PFS in endometrial carcinoma. The cutoff of MTV and TLG in PET/CT assessment could be an independent prognostic factors to predict aggressive forms of EC.

**Graphic Abstract:**

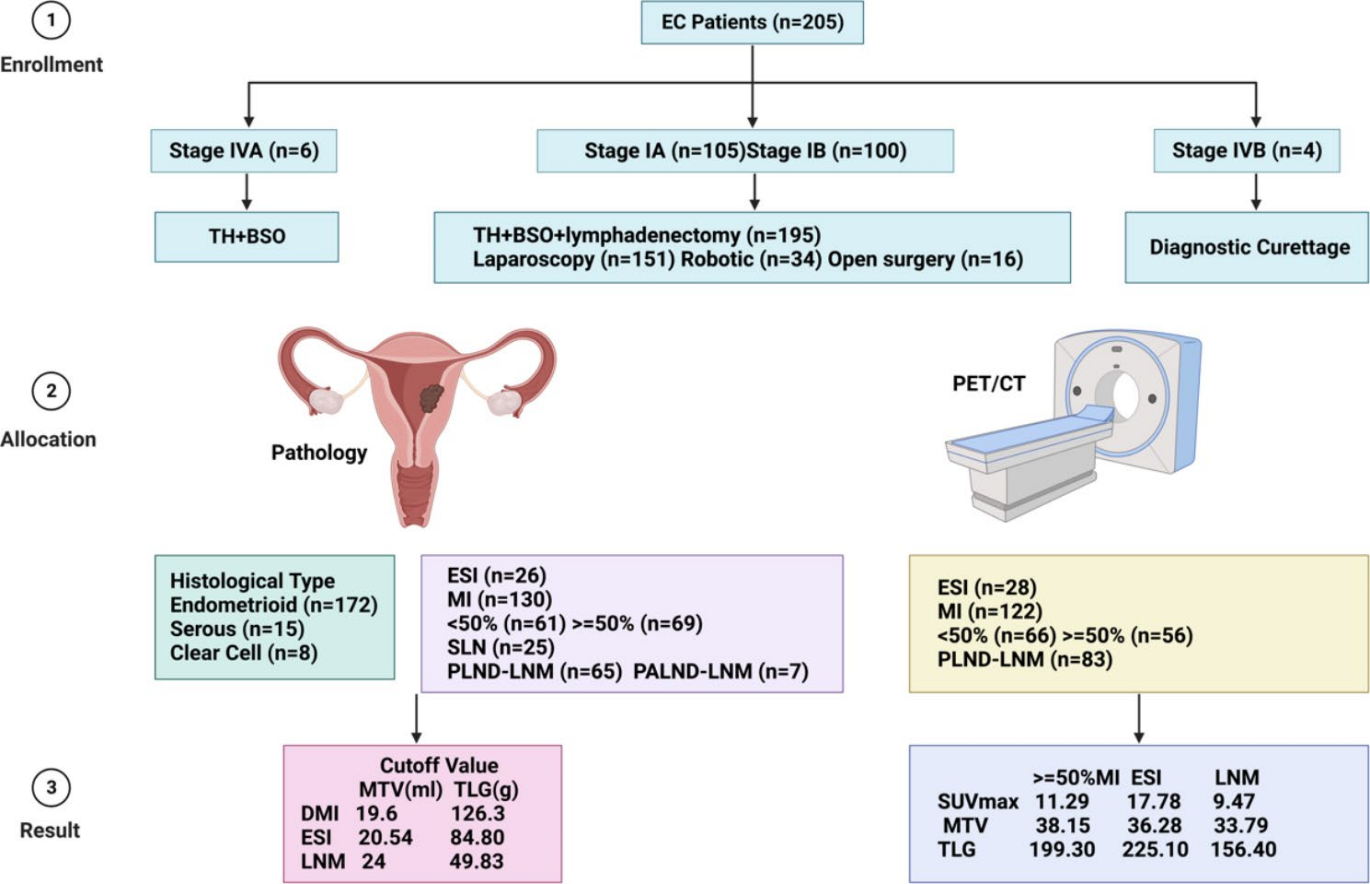

**Supplementary Information:**

The online version contains supplementary material available at 10.1186/s13014-023-02382-6.

## Introduction

There would be 66, 200 new endometrial cancer (EC) cases in 2023, with an estimated13,030 female deaths [1], with 250 more cases and 480 more deaths than in 2022. The 5-year progression free survival (DFS) gradually decreased in EC patients with stage I/II, III/IV, recurrence, and metastasis: 74.2–90.8%, 57.3–66.2%, 20.1–25.5%, and 16% [2]. According to the NCCN guidelines, patients with high-risk prognostic factors, including deep myometrial invasion (DMI) or endocervical stroma invasion (ESI) [3], should undergo systemic pelvic lymphadenectomy and removal of any enlarged or suspected para-aortic lymph node (PALN) [4]. Although accurately identifying the location of metastatic lymph nodes is essential to limit the surgical resection rate and avoiding excessive resections, for patients with early well-differentiated endometrial adenocarcinoma, the Gynecology Oncology Group (GOG) study showed that the overall risk of pelvic and para-aortic lymph node metastasis was 1–9% [5, 6], and routine systemic lymphadenectomy did not significantly improve survival but did increase the incidence of complications [7]. On the other hand, for patients undergoing fertility-sparing treatment, who undergo systematic evaluation by preoperative imaging for whether the tumor is limited to the endometrium, the existence of DMI and ESI has become particularly important [8].

The 2023 NCCN guidelines [4] and the American College of Radiology Imaging Network (ACRIN) suggested imaging scans to assist the diagnosis and treatment of endometrial cancer, since the metabolic abnormalities of tumors usually occur earlier than morphological changes; thus, morphological imaging methods like computed tomography (CT) and contrast-enhanced (CE) magnetic resonance imaging (MRI) have limitations in distinguishing whether the local morphological abnormality came from postoperative or postradiotherapy scars or tumor recurrence or from the omission of small metastatic lesions, resulting in false negative diagnoses or in judging lymph nodes as reactively enlarged, causing false-positives diagnoses [9, 10]. Interpreted whole-body ^18^fluorodeoxyglucose (^18^F)-fluoro-deoxy-glucose (FDG) positron emission tomography (PET) combined with computed tomography (CT) (^18^F-FDG-PET/CT) scanning is a molecular function imaging technology based on the glucose metabolism of tissue cells using the principle of positron radioactive tracing. It could be proposed for staging endometrial cancer, achieving high-sensitivity metabolic functional imaging with PET and high-resolution anatomical morphology imaging with CT [11].

The quantitative metabolic parameter standardized uptake value (SUV) was normalized by a region of interest (ROI) to patient weight, which represents the distribution of tracer uptake. The maximum standardized uptake value (SUVmax) represents the highest metabolic activity point in the tumor [12]. Generally, SUVmax ≥ 2.5 is an independent prognostic risk factor for malignant lesions [13]. However, the accuracy of SUV for the detection of lymph node metastasis (LNM) is controversial since ^18^F-FDG is not a specific tracer for malignant tumors; it is absorbed and metabolized by granulation tissue or macrophages during inflammation [14]. Other volumetric metabolic parameters, such as metabolic tumor volume (MTV) and total lesion glycolysis (TLG), are used to measure the total metabolic tumor burden of the patient in the entire tumor volume [15]. Both can generate the optimal cutoff point of imaging parameters through the receiver operator characteristic curve (ROC).

While the sensitivity and specificity of conventional PET/CT to detect positive lymph nodes were 72.3% and 92.9%, they were 95.7% and 95.4% for revealing extrauterine metastatic disease, respectively [16–18], with results possibly affected by lesion size, diffuse distribution, differentiation degree and survival of tumor cells [19]. Thus, it is difficult to form a precise standard cutoff point for PET/CT prediction parameters. The primary objective of the current study was to retrospectively analyze the predictive ability of three metabolic tumor parameters (SUVmax, MTV and TLG) from ^18^F-FDG PET/CT and to explore the best cutoff value as a prediction model to estimate prognostic factors of endometrial cancer, including DMI, ESI, and LNM, to accurately diagnose clinical-pathological staging.

## Materials and methods

### Study subjects and inclusion criteria

A total of 205 patients with pathologically proven diagnoses of endometrial cancer with no fertility-sparing desire were enrolled in this study from January 2018 to December 2021. The histological subtype complies with the 5th edition World Health Organization (WHO) Women Reproductive organ tumor classification [20]. All patients of EC underwent classical surgical staging by abdominal or minimally invasive surgical approach hysterectomy (TH) / bilateral salpingo-oophorectomy (BSO) and with or without surgical staging including pelvic lymphadenectomy or para-aortic lymphadenectomy under general anesthesia by gynecological oncologists. A minimally invasive operation was performed with traditional or robotic assistance laparoscopy, and para-aortic lymphadenectomy was conducted on patients with high-risk endometrial cancer. Another 42 postmenopausal women suspected endometrial cancer by curettage and underwent TH/BSO while postoperative pathological diagnosis confirmed as endometrial atypical hyperplasia (EAH) were included as control group. This retrospective study was approved by the institutional review board (IRB).

### Exclusion criteria

Patient treated with neo-adjuvant therapy or patients who are unable to undergo TH/BSO and surgical staging due to severe internal and external diseases: [1] Renal function damage; [2] Adrenal dysfunction; [3] Deep vein thrombosis or pulmonary embolism, thrombotic heart valve disease or thrombotic heart rhythm disease; [4] Inherited or acquired hypercoagulable diseases; [5] Serious cerebrovascular disease and stroke; [6] Severe coronary heart disease or myocardial infarction; [7] Uncontrolled hypertension; [8] Severe diabetes with vascular disease.

### Study setting

Whole-body ^18^F-FDG PET/CT was performed within 2 weeks of surgery in all patients before undergoing surgery. EC Patients were staged according to the FIGO 2009 criteria (Supplementary Table 1). The primary tumor lesion, histological subtype and grade, lymph-vascular space invasion (LVSI), depths of MI, ESI, and pelvic LNM were evaluated by two independent pathologists. Myometrial invasion less than 50% were defined as superficial myometrial invasion (SMI), while more than 50% was defined as deep myometrial invasion (DMI). ESI was regarded as invasion of cervical stroma. Clinical data with demographic characteristics (e.g., age, menopausal status, BMI) were recorded. Patient follow-up data have been collected from patient records individualized with the responsible gynecologists. Standard-of-care follow-up is clinical examinations quarterly during the first 2 years and biannually until 5 years after primary diagnosis. Progression was defined as local recurrence/progression in the pelvis or new metastases in the abdomen or at distant locations. For overall survival (OS), time was calculated as time from surgery to death. For disease free survival (DFS), time was calculated from surgery to recurrence or death.

### ^18^F-FDG PET/CT imaging protocol and image analysis

Whole-body ^18^F-FDG PET/CT was performed on a GE Discovery STE16 True Point scanner, with the scan range covering the skull base to the mid-thigh (General Electric Company, GE, USA). All patients fasted for at least 6 h prior to scanning and had an i.v. injection of 0.1 mCi ^18^F-FDG per kg of body weight. The required serum blood glucose ≤ 200 mg/dL. Patients drank 1.5 L of oral contrast agent and emptied their bladder during the 50-minute waiting period. PET/CT precisely scanned from the orbits to proximal thighs based on individual patient parameters. The PET protocol comprised five to six bed positions (3 min each) with a 20-min duration and was then reconstructed with CT images.

Maximum intensity projection and cross-sectional images after a 50-min uptake period were used to evaluate PET/CT images on the Segami Oasis workstation. Two independent radiological physicians, blinded to the clinical or pelvic MRI findings, made a diagnosis of the position of the primary tumor and tumor extension into the myometrium, cervical stroma, adnexa, vagina, urinary bladder or rectum mucosa as well as pelvic lymph nodes on PET/CT images, and a score of 1–4 indicated no disease to definite disease.

### ^18^F-FDG-PET/CT imaging parameters

Imaging parameters including lesion of the primary EC tumor, depth of MI, ESI, and LNM were assessed using bidimensional measurements. Volumetric parameters such as metabolic tumor volume (MTV) and total lesion glycolysis (TLG) were calculated. MTV is a volumetric measurement by semiautomatic boundary delineation methods, calculated by volume of interest (VOI), using a specific threshold value of SUV > 2.5. TLG is defined as the product of the mean standardized uptake value (SUVmean) and MTV. Optimal cutoff values for MTV and TLG were identified from the receiver operating characteristic (ROC) curves using the Youden index for predicting DMI, ESI and LNM. ^18^F-FDG avidity of the tumor was assessed using metabolic parameters as the SUVmax and the mean standardized uptake value (SUVmean) measurements based on SUV levels in healthy background tissue. SUVmax was calculated by tissue radioactivity (injection dose or weight of subject), while increased ^18^F-FDG uptake with SUVmax ≥ 2.5 was defined as tumor lesion and metastasis. Furthermore, the sensitivity, specificity, positive predictive value (PPV), negative predictive value (NPV), and false-negative rate (FNR) were used for the detection of prognostic indicators. The prognostic value of the imaging parameters was explored using the Kaplan-Meier with log-rank test with hazard ratios (HRs) by OS and DFS.

### Statistical analysis

Analyses were performed in SPSS 27.0 (IBM Corp. SPSS Inc, Armonk, NY, USA). Descriptive statistics of cutoff values, maximum SUV, mean SUV, MTV, and TLG were recorded. Distributed continuous and categorical data were generated by T tests, Fisher, Fisher’s exact tests and chi-square tests and are reported as the means and SD. Abnormally distributed data were analyzed by the Mann–Whitney U test and Kruskal–Wallis test and are reported as the median and range. OS, DFS and HR were analyzed by the Kaplan Meier and Cox regression. The statistical significance of differences in DMI, ESI and LNM as determined by PET/CT sensitivity, specificity, accuracy, PPV and NPV were compared by McNemar’s test. The association between SUVmax, MTV and TLG in DMI, ESI and LNM was analyzed by Pearson correlation with a 95% exact binomial confidence interval (CI). A P value less than 0.05 generated by two-sided tests was considered statistically significant.

## Results

### Demographics and patient treatment

A total of 205 patients with endometrial cancer were enrolled in the current study between January 2018 to December 2021. All patients were treated according to the NCCN guidelines for endometrial cancer. The patients’ demographic characteristics are shown in Table 1. Mean (range) follow-up time for survivors was 51.05 ± 5.00 (23–61) months and date of last follow-up was June 2023. The mean age was 58 years (35–87 years), and the mean BMI was 24.69 kg/m^2^ (range, 18.20–30.00 kg/m^2^). According to the 2009 International Federation of Gynecology and Obstetrics (FIGO) criteria, 4 additional IVB patients underwent diagnostic curettage due to widespread liver, lung and bone metastasis. The histological diagnoses of these four patients were based on uterine biopsies and recorded FIGO stage on findings from diagnostic imaging. Apart from 6 IVA patients, including 5 with bladder mucosa metastasis, 2 patients with bowel mucosa metastasis underwent TH/BSO, and the remaining 195 (95.12%) patients underwent primary surgical resection with TH/BSO plus sentinel/pelvic/para-aortic lymphadenectomy. Regarding the surgical approach, a total of 185 patients (90.24%) underwent minimally invasive surgery—laparoscopy in 73.66% (151/205) and robotic in 16.59% (34/205)—and 16 patients (7.80%) underwent open surgery. Retroperitoneal para-aortic lymph node dissection was performed either via a laparoscopic approach and robotics platform or open surgery. Adjuvant therapy was given in 50.73% (104/205) of the patients, including radiation in 50.73% (104/205), chemotherapy in 40.98% (84/205), and hormonal treatment in 5.85% (12/205) of the patients.

**Table 1:** Patient characteristics and surgicopathological findings in 205 endometrial cancer patients

Demographic characteristics	Results
Age, mean (range)	58 (35–87)
BMI, mean (range)	24.69 (18.20–30.00)
Postmenopausal, n (%)	131 (63.90%)
Treatment	
Hysterectomy and bilateral salpingooophorectomy	6 (2.93%)
Sentinel lymphadenectomy	25 (12.20%)
Pelvic lymphadenectomy	131 (63.90%)
Para-aortic lymphadenectomy	39 (19.02%)
diagnostic curettage	4 (4.88%)
Surgical approach	
Open surgery	16 (7.80%)
Minimally invasive surgery	185 (90.24%)
Laparoscopy	151 (73.66%)
Robotic	34 (16.59%)
Adjuvant therapy	104 (50.73%)
Radiotherapy	104 (50.73%)
Chemotherapy	84 (40.98%)
Hormonal	12 (5.85%)
2009 FIGO stage, n (%)	
Stage IA	102 (49.75%)
Stage IB	21 (10.24%)
Stage II	13 (6.34%)
Stage IIIA	9 (4.39%)
Stage IIIB	7 (3.41%)
Stage IIIC1	36 (17.56%)
Stage IIIC2	7 (3.41%)
Stage IVA	6 (2.93%)
Stage IVB	4 (1.95%)
2009- (convert to) 2023 FIGO	
IA-IA1	48
IA-IA2	42
IA-IC	2
IA-IIB	3
IA-IIC	6
IB-IB	10
IB-IIC	11
II-IIA	10
II-IIB	1
II-IIC	3
IIIA-IIIA1	8
IIIA-IIIA2	1
IIIB-IIIB1	7
IIIB-IIIB2	1
IIIC1-IIIC1i	32
IIIC1-IIIC1ii	4
IIIC2-IIIC2i	6
IIIC2-IIIC2ii	1
IVA-IVA	6
IVB-IVB	4
Mean follow-up period	
Progression	23(11.22%)
Follow-up Metastatic Site	
Bladder mucosa	4
Bowel mucosa	3
Pelvic Lymph nodes	2
Para-aortic lymph nodes	3
Liver	1
Lung	7
Bone	3
Histologic subtype, n (%)	
Endometrioid	172 (88.78%)
Non-endometrioid	23 (11.22%)
serous	15 (7.32%)
clear cell carcinoma	8 (3.90%)
Histologic grade, n (%)	
Grade 1	90 (43.90%)
Grade 2	52 (25.37%)
Grade 3	63 (30.73%)
LVSI	75 (36.59%)
Myometrial invasion, n (%)	
< 50%	61 (29.76%)
≥ 50%	69 (33.66%)
Cervical stroma invasion, n (%)	
No	177 (86.34%)
Yes	26 (12.68%)
Pelvic Lymph node metastases, n (%)	
No	66 (50.38%)
Yes	65 (49.62%)
Paraaortic lymph node metastases, n (%)	
No	32 (82.05%)
Yes	7 (17.95%)

The histological subtype complies with the 4th edition World Health Organization (WHO) Women Reproductive organ tumor classification, 2020. Histologic grade was divided into Grade 0: Grade cannot be assessed, Grade 1, G1: Well differentiated, Grade 2, G2: Moderately differentiated, Grade 3, G3: Poorly differentiated or undifferentiated

### Surgico-pathological features

On final pathology, 105 patients (49.75%) were diagnosed with early stage IA, and the remaining 100 patients were diagnosed with stage IB, 21 (10.24%) and higher: stage II: 13 (6.34%), stage III: 59 (28.78%), and stage IV: 10 (4.88%). LVSI was depicted in 36.59% of patients (75/205). The percentages of tumor differentiation divided into grade 1, 2, and 3 tumors were 43.90% (90/205), 25.37% (52/205), and 30.73% (63/205), respectively. The most common histological type was endometrioid (172, 88.78%), followed by serous (15/205, 7.32%) and clear cell carcinoma (8/205, 3.90%). MI was detected in 29.76% (61/205) or 33.66% (69/205) of patients. ESI was observed in 12.68% (26/205) of patients. Pelvic lymph node dissection (PLND) was performed in 131 patients (95%, 131/205) and revealed LNM in 49.62% (65/131) of the patients, while paraaortic lymph node dissection (PALND) was conducted in 39 patients (19.02%) and revealed paraaortic LNM in 17.95% (7/39). Another 25 patients (12.20%) underwent sentinel lymphadenectomy (SLN). (Table 1).

### Re-categorized according to 2023 FIGO staging

In 2023, the new FIGO staging system incorporates comprehensive clinical, surgical, histopathological, TNM (Tumor, Lymph Node, Metastasis), prognostic high-risk factors, and molecular subtyping for staging EC [21]. We re-categorized patient previously staged according to the 2009 FIGO system according to 2023 FIGO staging system. Since 2023 FIGO EC Staging, non-aggressive histological types are only composed of low-grade (grade 1 and 2) EECs, other types like high-grade EECs (grade 3), serous or clear cell were all defined as aggressive histological types. Furthermore, LVSI were also distinguished with no/focal or extensive/substantial. Thus, the adjustment of the EC Staging from 2009 to 2023 were: IA-IA1(N = 48), IA-IA2 (N = 42), IA-IC (N = 2), IA-IIB (N = 3), IA-IIC (N = 6), IB-IIC (N = 11). (Table 1) (Supplementary Table 1).

### OS and DFS

**Fig. 1:**
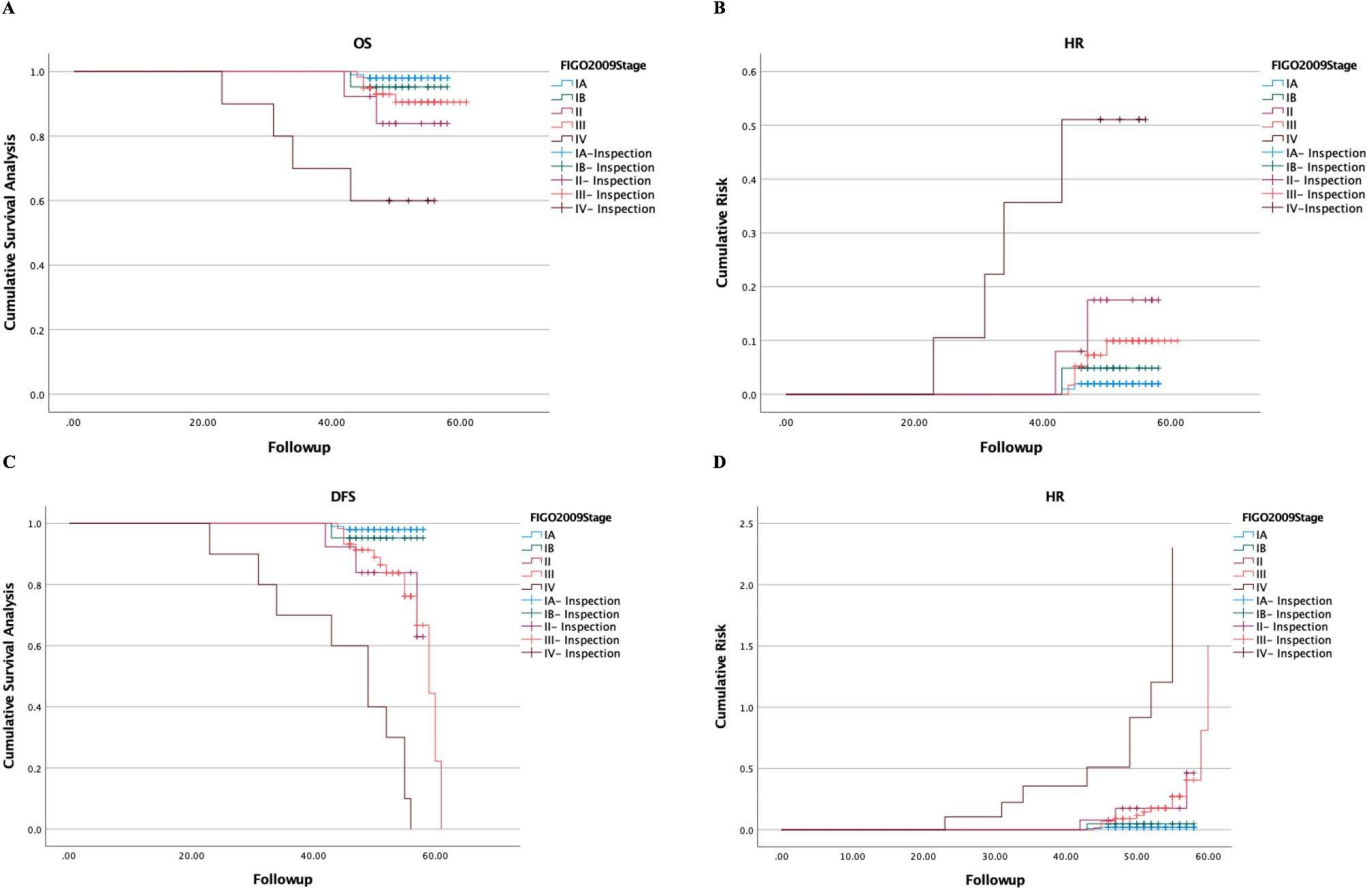
OS and DFS according to 2009 EC FIGO Staging. **(A)** Cumulative survival analysis of OS; **(B)** Cumulative Risk of HR for OS; **(C)** Cumulative survival analysis of PFS; **(D)** Cumulative Risk of HR for PFS.

Kaplan-Meier estimated 4-year OS (HR) for Stage IA, IB, II, III and IV according to 2009 FIGO was 98.00% (0.22), 95.20% (0.04), 83.90% (0.18), 90.50% (0.09) and 60% (0.51). Accordingly, estimated 4-year DFS (HR) for the stage IA-III was 98% (0.02), 95.20% (0.05), 76.90% (0.27) and 76.30% (0.35), all the patients in stage IV occurred recurrence and progression (Fig. 1).

### Association between PET/CT Tumor parameters and surgico-pathological features

Upon comprehensive PET/CT analysis of and review of the 205 patients’ histopathological results, 83 patients (40.49%) had LNM, 28 patients (13.66%) had ESI, and 122 patients (59.51%) had MI, among SMI was detected in 32.20% (66/205) and 27.32% (56/205) of patients. There was a significant difference between DMI, ESI and pelvic LNM and SUVmax, SUVmean, MTV and TLG (*P* < 0.01). Additionally, a significant association between SUVmax vs. MTV and SUVmax vs. TLG in DMI (*R* = 0.2896, *P* = 0.0304; *R* = 0.4611, *P* = 0.0003), ESI (*R* = 0.4441, *P* < 0.0001, *R* = 0.4793, *P* < 0.0001) and LNM (*R* = 0.6352, *P* < 0.0001, *R* = 0.7734, *P* < 0.0001) was observed (Table 2).

**Table 2:** Parameters of diagnostic performance on a per-patient basis

Surgico-pathological features		MTV vs. SUVmax	TLG vs. SUVmax
DMI	Pearson r	0.2896	0.4611
	95% CI	0.02893 to 0.5134	0.2256 to 0.6457
	P	0.0304	0.0003
ESI	Pearson r	0.4441	0.4793
	95% CI	0.3435 to 0.8151	0.5626 to 0.8897
	P	< 0.0001	< 0.0001
LNM	Pearson r	0.6352	0.7734
	95% CI	0.2526 to 0.6021	0.2940 to 0.6299
	P	< 0.0001	< 0.0001

DMI: Deep Myometrial invasion, ESI: Endocervical stroma invasion, LNM: Lymph node metastases

### Difference of metabolic parameters in PET/CT between EC and EAH patients

Next, we compared metabolic parameters in PET/CT between EC and EAH patients’ myometrium, SUVmax (7.04 vs. 1.81), SUVmean (3.32 vs. 1.22), MTV (27.74 vs. 14.67), TLG (106.60 vs. 17.92), endocervical stroma: SUVmax (4.04 vs. 1.29), SUVmean (2.07 vs. 0.77), MTV (16.29 vs. 11.52), TLG (47.01 vs. 8.88), and lymph node SUVmax (4.85 vs. 1.06), SUVmean (2.68 vs. 0.72), MTV (23.22 vs. 6.95), TLG (77.03 vs. 5.05). There was a significant difference in metabolic parameters in PET/CT on myometrium, endocervical stroma and lymph node between EC and EAH patients (*P* < 0.01) (Table 3).

**Table 3:** Difference in metabolic parameters in PET/CT on myometrium, endocervical stroma and lymph node between EC and EAH patients

		SUVmax	SUVmean	MTV	TLG	*p*-value
Myometrium	EC	7.04 ± 0.34	3.32 ± 0.12	27.74 ± 0.67	106.60 ± 5.43	< 0.01
	EAH	1.81 ± 0.08	1.22 ± 0.06	14.67 ± 0.52	17.92 ± 1.20	
Endocervical stroma	EC	4.04 ± 0.38	2.07 ± 0.12	16.29 ± 0.64	47.01 ± 5.24	< 0.01
	EAH	1.29 ± 0.05	0.77 ± 0.04	11.52 ± 0.54	8.88 ± 0.63	
Lymph node	EC	4.85 ± 0.30	2.68 ± 0.13	23.22 ± 0.73	77.03 ± 5.42	< 0.01
	EAH	1.06 ± 0.02	0.72 ± 0.04	6.95 ± 0.27	5.05 ± 0.35	

### Prediction of myometrial and endocervical stromal invasion and regional lymph node metastasis by preoperative imaging parameters

#### MI detection

**Fig. 2:**
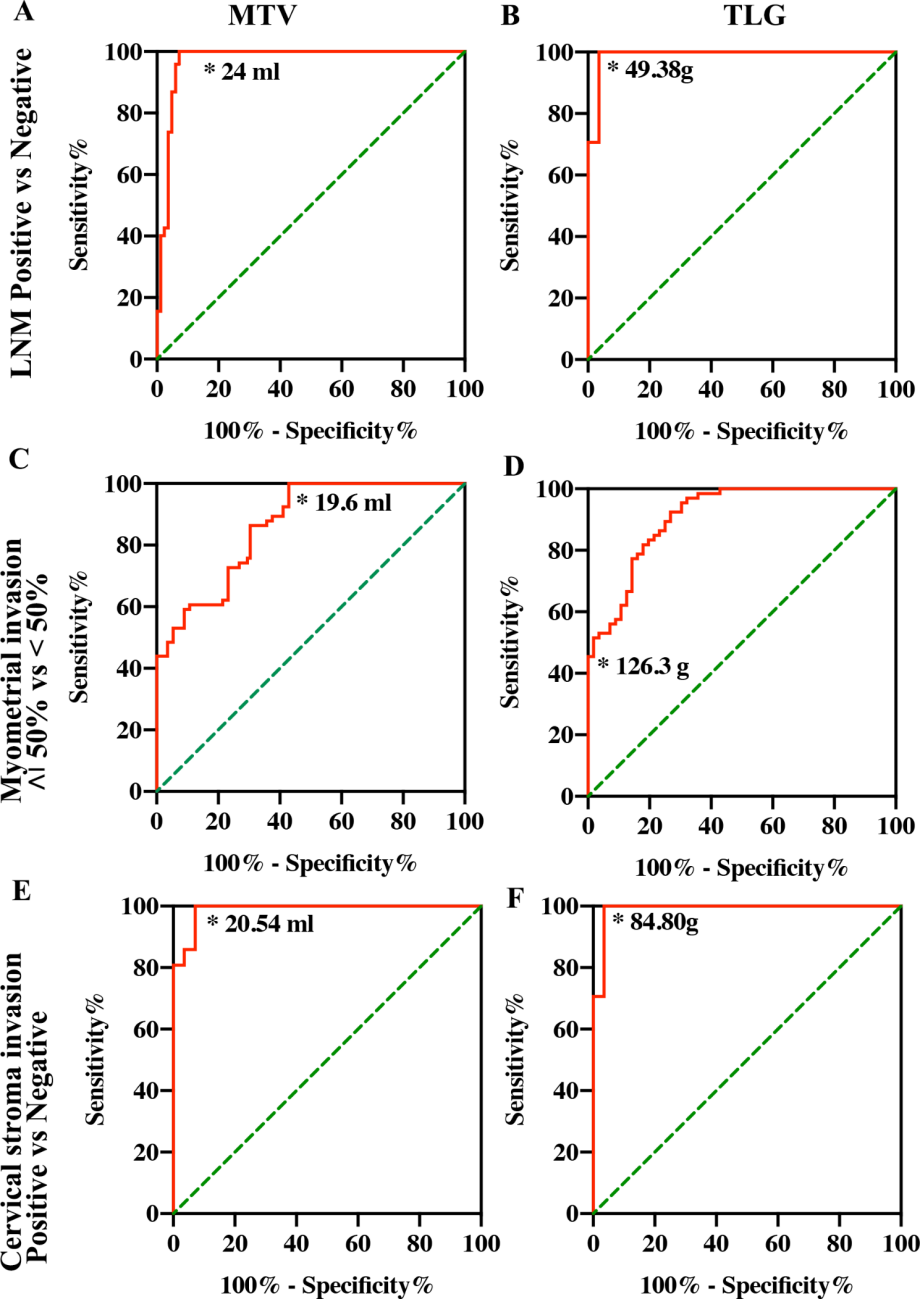
Lymph node metastasis (LNM) positivity and negativity calculated by PET/CT based on ROC curve analysis in 205 patients with endometrial cancer **(A).** MTV **(B).** TLG, myometrial invasion ≥ 50% and < 50% **(C).** MTV, **(D).** TLG, cervical interstitial invasion **(E)**. MTV, **(F).** Best cutoff point of TLG; ordinate, sensitivity, abscissa, specificity;

The mean SUVmax and SUVmean of MI patients were significantly higher than those of patients who did not have MI (SUVmax value 10.75 vs. 1.58, SUVmean value 4.59 vs. 1.46, *P* < 0.01). The mean SUVmax and SUVmean of DMI patients were significantly higher than SMI patients (SUVmax value 11.29 vs. 10.29, SUVmean value 5.20 vs. 4.08, *P* < 0.01). The mean MTV and TLG values of patients who had myometrial invasion and did not have invasion were 34.68 vs. 17.55 ml and 161.60 vs. 25.71 g, respectively. The MTV and TLG cutoff values for MI were calculated to be 25.51 ml and 70.74 g, respectively, with a sensitivity and specificity yield of 100% (AUC = 1, *P* < 0.01). The mean MTV and TLG values of patients who had SMI and DMI 50% were 31.73 vs. 38.15 ml and 199.30 vs. 129.60 g, respectively. ROC curve analysis calculated an MTV cutoff value of 19.6 ml for identifying SMI and DMI (AUC = 0.8643, sensitivity = 43.94%, specificity = 100%, 95% CI 0.8025–0.9261, *P* < 0.01). The TLG cutoff value was 126.3 g for SMI and DMI (AUC = 0.9099, sensitivity = 45.45%, specificity = 100%, 95% CI 0.8605–0.9593, *P* < 0.01) (Fig. 2) (Table 4) (Supplementary Table 2).

**Table 4:** ^18^ F-FDG PET/CT tumor markers in relation to surgical and histological tumor characteristics in 205 endometrial cancer patients

Parameters	Myometrial invasion		*P*	*t*	Myometrial invasion		*P*	*t*	Endocervical stromal involvement		*P*	*t*	Lymph node metastases		*P*	*t*
	No	Yes			< 50%	≥ 50%			No	Yes			No	Yes		
No	83	122			66	56			177	28			122	83		
SUVmax	1.58 ± 0.07	10.75 ± 0.21	< 0.01	34.59	10.29 ± 0.23	11.29 ± 0.36	< 0.05	2.383	1.92 ± 0.04	17.38 ± 0.65	< 0.01	54.12	1.72 ± 0.04	9.47 ± 0.34	< 0.01	27.41
SUVmean	1.46 ± 0.06	4.59 ± 0.09	< 0.01	26.45	4.08 ± 0.08	5.20 ± 0.12	< 0.01	7.659	1.43 ± 0.04	6.12 ± 0.19	< 0.01	35.23	1.44 ± 0.04	4.49 ± 0.16	< 0.01	21.27
MTV(ml)	17.55 ± 0.48	34.68 ± 0.45	< 0.01	25.46	31.73 ± 0.42	38.15 ± 0.55	< 0.01	9.378	13.13 ± 0.29	36.28 ± 1.51	< 0.01	24.77	16.03 ± 0.37	33.79 ± 0.85	< 0.01	21.34
ROC Area	1		< 0.01		0.8643				0.9881				0.9705			
Std.Error	0.00				0.03153				0.008541				0.01449			
95% CI	1	1			0.8025–0.9261		< 0.01		0.9714-1.000		< 0.01		0.9421–0.9989		< 0.01	
Cutoff					19.6				20.54				24			
Sensitivity					43.94				100				100			
Specificity					100				92.86				92.77			
TLG	25.71 ± 1.34	161.60 ± 4.57	< 0.01	24.05	129.60 ± 3.21	199.30 ± 6.14	< 0.01	10.50	18.84 ± 0.76	225.10 ± 11.58	< 0.01	41.86	23.02 ± 0.89	156.40 ± 7.06	< 0.01	22.55
ROC Area	1				0.9099				0.9895				0.9642			
Std.Error	0.00				0.02520				0.01044				0.01771			
95% CI	1	1			0.8605–0.9593		< 0.01		0.9690-1.000				0.9294–0.9989		< 0.01	
Cutoff					126.3				84.80				49.38			
Sensitivity					45.45				100				100			
Specificity					100				96.43				93.98			
Pathology	75	130			61	69			179	26			140	65		
TP		122			61	56				26				65		
TN		75			139	136				177				122		
FP		0			5	0				2				18		
FN		8			0	13				0				0		
Sensitivity		93.85			100	81.16				100				100		
Specificity		100			96.53	100				98.89				87.14		
Accuracy		96.1			97.56	93.66				99.02				91.22		
PPV		100			92.42	100				92.85				78.31		
NPV		90.36			100	91.28				100				100		

TP, true positive; TN, true negative; FP, false positive; FN, false negative; sensitivity; specificity; accuracy; PPV, positive predictive value; NPV, negative predictive value

*p* < 0.05 (2-tailed)

PET/CT understaged MI in 8 patients and DMI in 13 patients, and it over-staged SMI in 5 patients. Parameters of diagnostic performance including true positive (TP), true negative (TN), false-positive (FP), false negative (FN), sensitivity [(TP/TP + FN)*100%], specificity [(TN/FP + TN)*100%], accuracy [(TP + TN)/N*100%], positive predictive value (PPV) [(TP/TP + FP)*100%], and negative predictive value (NPV) [(TN/FN + TN)*100%] of PET/CT on a per-patient basis are shown in Table 4. The sensitivity of PET/CT to detect MI was 93.85%, and the specificity was 100%, with an accuracy of 96.10% with a PPV of 100% and an NPV of 90.36%. The sensitivity of PET/CT to detect SMI and DMI was 100% vs. 81.16%, and the specificity was 96.53% vs. 100%, accuracy 97.56% vs. 93.66%. The PPV and NPV were 92.42% vs. 100% and 100% vs. 91.28%, respectively (Fig. 2) (Table 4) (Supplementary Table 2).

#### Endocervical stroma invasion (ESI)

The mean SUVmax and SUVmean in patients who had ESI were significantly higher than those in patients who did not (SUVmax value 17.38 vs. 1.92, SUVmean value 6.12 vs. 1.43, *P* < 0.01). The mean MTV and TLG of patients who had ESI and did not have ESI were 36.28 vs. 13.13 ml and 225.10 vs. 18.84, respectively (*P* < 0.01). Using MTV cutoff values for predicting ESI, MTV > 20.54 ml yielded significantly higher specificity (100%) and specificity (92.86%). The TLG cutoff value was 84.80 g for ESI (AUC = 0.9895, sensitivity = 100.00%, specificity = 96.43%, 95% CI 0.9690-1.000, *P* < 0.01). PET/CT over-staged ESI in 2 patients. The sensitivity of PET/CT to detect ESI was 100%, the specificity was 98.89%, and the accuracy was 99.02%. The PPV and NPV were 92.85% and 100.0%, respectively (Fig. 2) (Table 4) (Supplementary Table 2).

#### Pelvic lymph node Metastasis (LNM)

**Fig. 3:**
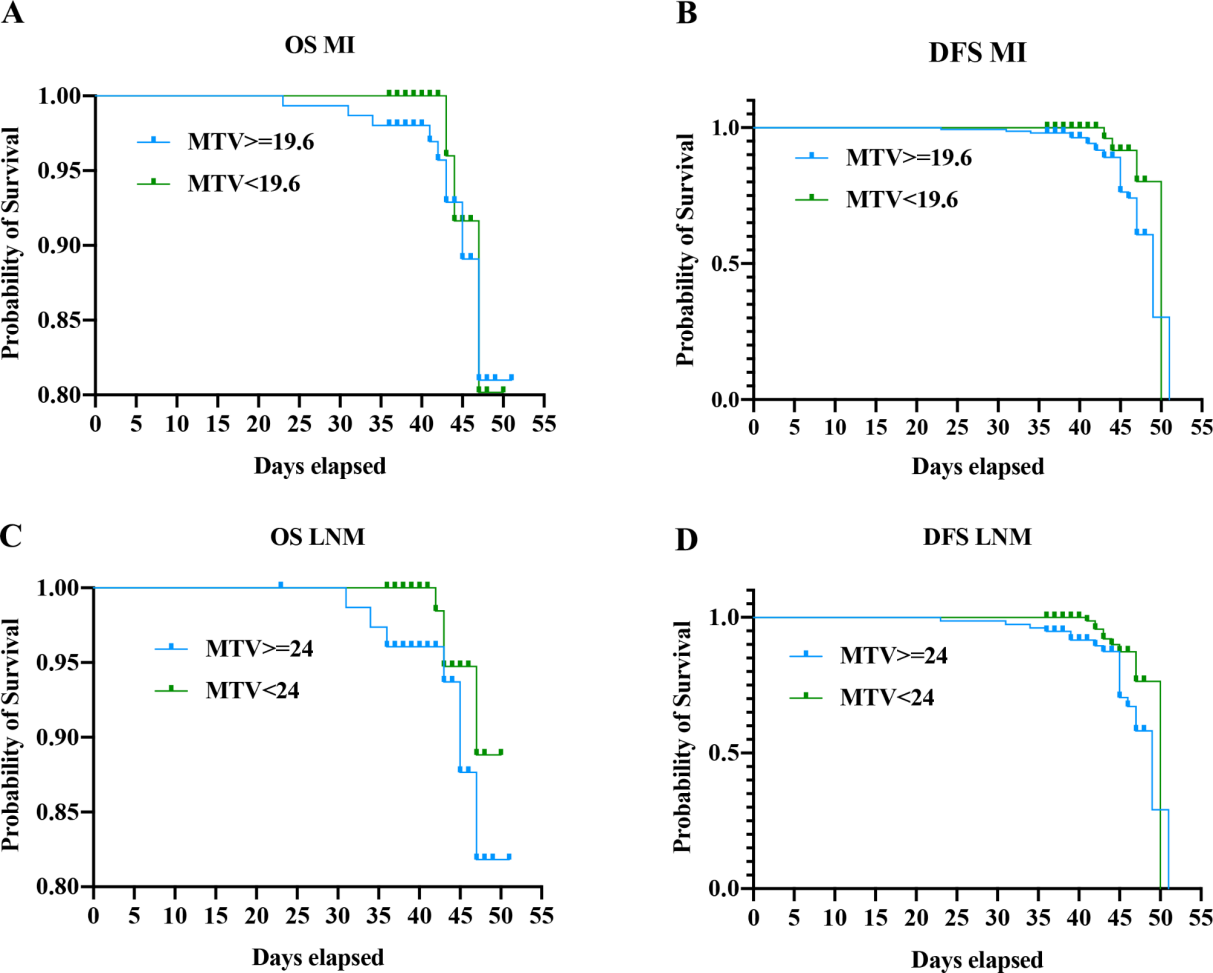
Survival of OS and DFS in MTV of MI and LNM. OS in MTV of PET/CT; **(B).** DFS in MTV of PET/CT; **(C).** OS of PET- positive LN with MTV; **(D).** DFS of PET- positive LN with MTV.

For patients with negative and positive pelvic lymph nodes, the SUVmax and SUVmean were 1.72/1.44 vs. 9.47/4.49, respectively, and the mean MTV was 16.03 and 33.79, respectively. MTV yielded a cutoff value for pelvic lymph node positivity of 24 ml (AUC = 0.9705, sensitivity = 100%, specificity = 92.77%, 95% CI 0.9421–0.9989, *P* < 0.01). TLG was 23.02 and 156.40 between patients with negative and positive pelvic lymph nodes, respectively, and the TLG cutoff value was 49.38 g for lymph node positivity (AUC = 0.9642, sensitivity = 100.00%, specificity = 93.98%, 95% CI 0.9294–0.9989, *P* < 0.01). PET/CT over-staged the pelvic LNM in 18 patients. The sensitivity of PET/CT to detect pelvic LNM was 100%, the specificity was 87.14%, and the accuracy was 91.22%. The PPV and NPV were 78.31% and 100.0%, respectively (Figs. 2 and 3) (Table 4) (Supplementary Table 2).

### Prediction of progression-free survival by preoperative imaging parameters

**Fig. 4:**
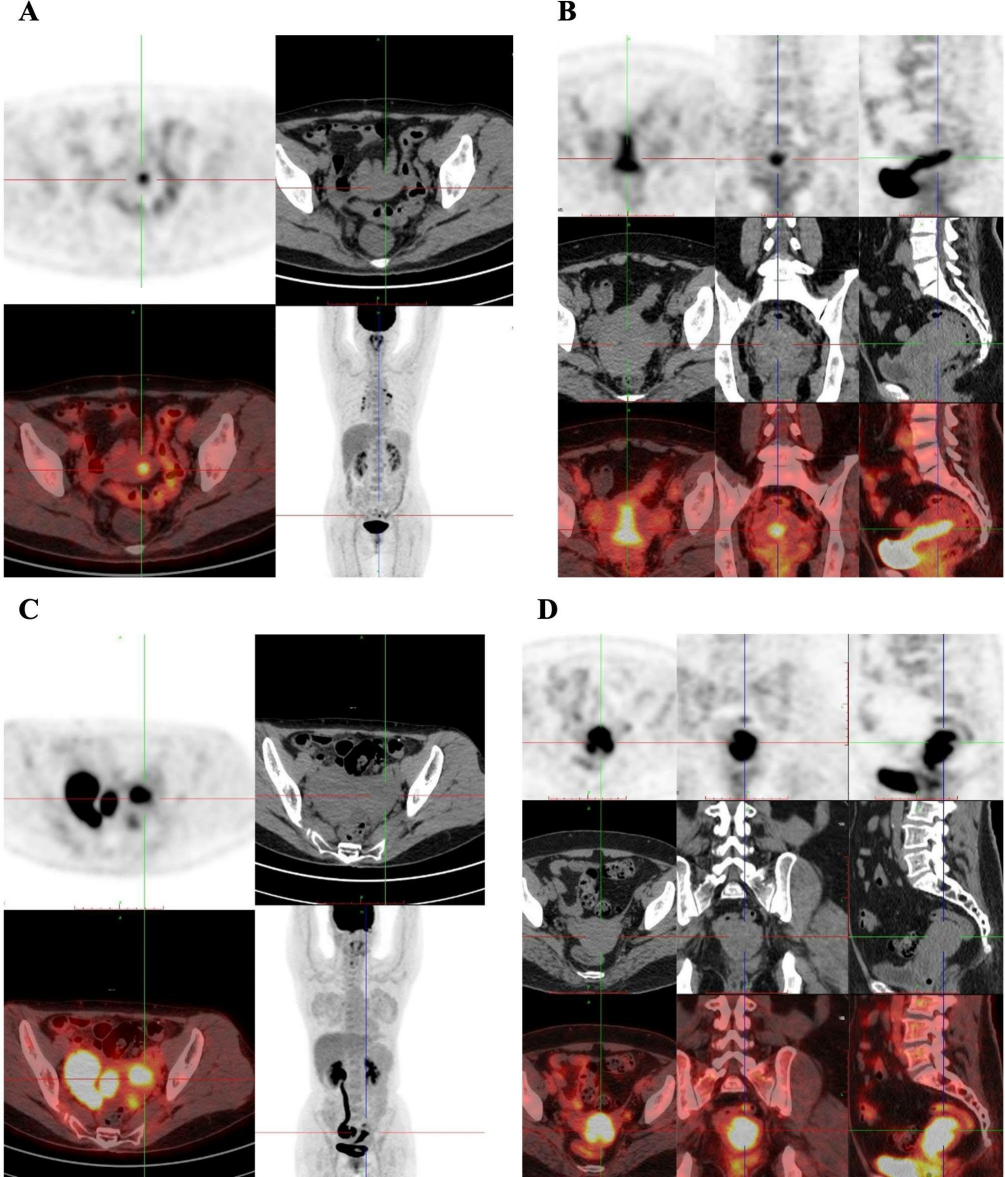
PET/CT images of endometrial cancer including trans-axial CT, fused PET/CT images of the lower abdomen revealed that intense 18 F-FDG uptake in the uterine and ovary cavity. **A.**, A 54-year-old female patient with histologically confirmed endometrial adenocarcinoma in FIGO IA Stage, intrauterine lesions SUVmax 5.5; **B.** A 47-year-old female patient with histologically confirmed endometrial adenocarcinoma in Stage IA, intrauterine lesions range 4.1 × 1.7 cm^2^ with SUVmax 8.3. **C.** A 47-year-old female patient with histologically confirmed endometrial adenocarcinoma in Stage II, lesions on right ovary is range 6.8 × 4.6 cm^2^ with SUVmax 18.9 and intrauterine lesions SUVmax 17.0. **D.** A 58-year-old female patient with histologically confirmed endometrial serous carcinoma, intrauterine lesions rang 2.8*2.0*4.9 cm ^3^ with SUVmax 15.5, along with endocervical stroma lesions diameter 2.3 cm and SUVmax 4.1

Patient follow-up was performed according to clinical examinations quarterly during the first 2 years and biannually until 5 years after primary diagnosis. The mean (range) follow-up time for survivors was 33 (20–44) months, and the date of last follow-up was 16 August 2021. In total, 14 out of the 205 (6.83%) patients experienced progression among patients primary staged as FIGO III–IV according to disease assessment adopts (Response Evaluation Criteria in Solid Tumor, RECIST, version 1.1). In total, 23 out of the 205 (11.22%) patients experienced progression, Among these patients’ metastatic lesions: bladder mucosa (n = 11), bowel mucosa (n = 4) 3, pelvic lymph nodes (n = 2), para-aortic lymph nodes (n = 3), liver (n = 1), lung (n = 7), bone (n = 3). (Table 1). A total of 5 patients suffered myocardial infarction and 2 died of pulmonary embolism. There was a relationship between cutoff of MTV and survival. When adjusting for preoperative high-risk status of MI, Patients with MTV > = 19.6 ml tended to predict poor OS and PFS with univariate hazard ratios (HRs) of 1.312 (*p* = 0.3766) and 2.210 (*p* = 0.0758), PET- positive LN with MTV cutoff > = 24 ml remained significantly associated with decreased DFS (HR = 2.178, *p* = 0.0065), and decreased OS (HR = 2.309, *p* = 0.0588), respectively (Fig. 4) (Supplementary Table 2).

## Discussion

PET/CT could evaluate patients with endometrial cancer before surgery and is currently an important reference for the establishment of surgical procedures and adjuvant therapy [22, 23]. PET/CT-derived parameters need to be confirmed in combination with preoperative metabolic and volumetric biomarkers for better prediction of high-risk factors [12]. The metabolic parameter SUVmax value with the highest metabolic activity point in the tumor was an independent prognostic factor for MI and lymph node positivity in ^18^F-FDG-PET/CT [24, 25]. Furthermore, the volumetric parameters MTV and TLG can demonstrate tumor volume burden, which can achieve more precise prognosis prediction than SUVmax. Previous differences in the diagnostic accuracy of preoperative SUV, MTV and TLG detected on whole-body PET/CT have been achieved for the different patient cohort studies of EC [26]. The median SUVmax with 95% CI of DMI, ESI, and LNM was 16.6(14.4–18.1), 15.9(12.3–19.0), and 17.9(12.1–25.0), while MTV was 26 [22–37], 27(16–43) and 43(28–101), respectively, based on a large population-based study with 215 and 287 EC patients [19, 27]. MD Anderson enrolled 108 EC patients to detect positive lymph nodes and peritoneal disease, and the specificity, specificity, PPV, NPV and FN of PET/CT were 45.8%, 91.1%, 61.1%, 84.7% and 54.2% vs. 37.5%, 97.8%, 75%, 90.0% and 62.5%, respectively. The median peak SUV, SUV3 and MTV were 15.9 vs. 12.5, 25.5 vs. 31.8, and 9.6 vs. 19.6, respectively, with negative and positive lymph nodes [28]. One multicenter study including 1431 patients in China revealed that the overall sensitivity, specificity, AUC and accuracy of PET/CT in detecting LNM were 0.68 (95% CI 0.63–0.73), 0.96 (95% CI 0.96–0.97), 0.82, and 0.75, respectively. The corresponding indices for detecting PALN metastasis were 0.70 (95% CI 0.58–0.79), 0.92 (95% CI 0.9–0.94), 0.84, and 0.77, respectively [29]. Our study was basically in line with previous studies, we identified a significant difference in PET/CT parameters of SUVmax, SUVmean, MTV, TLG on MI, ESI, and LNM between EC and EAH patients (*P* < 0.01).

Mapelli P [30] investigated 57 patients and concluded TLG 40-50-60 and MTV 60 of primary EC have prognostic value in discriminating FIGO. Maria Picchioa [31] focused on high-risk EC patients and concluded the overall PET/CT patient-based sensitivity, specificity, positive predictive value, negative predictive value and accuracy were 57.1, 100.0, 100.0, 86.4, and 88.5%, respectively. We demonstrated a significant relationship between metabolic parameters (SUVmax, SUVmean) and volumetric parameters (MTV, TLG) and MI, ESI, and LNM on PET/CT (*P* < 0.01) in 205 patients including all staging as well as histological type and grade. We detected SUVmax, SUVmean, MTV and TLG to predict DMI, ESI and pelvic LNM with sensitivity, specificity, accuracy, PPV, and NPV 100%/100%/100%, 96.53%/98.89%/87.14%, 97.56%/99.02%/91.22%, 92.42%/92.85%/78.31%, 100%/100%/100%, respectively.

Since the parameters SUVmax, MTV and TLG are affected by tumor volume, tissue uptake of ^18^F-FDG, tissue proliferation after radiotherapy, etc., using cutoff values to predict LNM and aggressive disease can be a comprehensive and valuable technique in EC treatment. Fasmer et al. calculated that MTV > 27 ml with an ROC curve yielded 70-74% specificity, 75% accuracy, and an odds ratio of12.2 and was significantly associated with reduced progression-free survival (HRs = 1.003, *p* < 0.01) based on a large population-based study [19]. Mehmet et al. summarized 44 EC patients and revealed MTV and TLG cutoff values yielding 19.6 ml and 90 g for early-stage EC, 14.3 ml and 173.4 g for MI, and 29.7 ml and 283.1 g for LNM, respectively. They also observed MTV was also observed as a significant prognostic factor for DFS time with an inverse proportion. Longer DFS time was observed in patients with lower MTV [32, 33].

Compared with the previous studies investigated cutoff values of MTV and TLG separately on metastatic site, we fully assessed the optimal cutoff values for MTV and TLG to predict DMI, ESI, and LNM preoperatively in EC patients based on a retrospectively large sample. The cutoff for MTV and TLG on DMI, ESI, and LNM in our study were 19.6 ml/126.3 g, 20.54 ml/84.80 g and 24 ml/49.83 g, which yielded significantly higher specificity (*P* < 0.01) and accuracy (*P* < 0.01) based on the ROC curves. Both MTV over 19.6 ml in DMI and over 24ml in LNM were significantly associated with decreased OS and DFS time. We expect to use the new observation of the above cutoff value as the risk model for endometrial cancer to judge high-risk factors in future prospective research.

In 2021, the European Society of Gynecological Oncology (ESGO) /European Society for Therapeutic Radiation Oncology(ESTRO)/European Society of Pathologists(ESP) [34] recommend ProMisE typing molecular testing for all EC patients: p53 abnormality (p53 abn), POLE exonuclease domain mutation (POLE EDM, or POLE mut), mismatch repair deficient (MMRd) and non-specific molecular profile (NSMP) [35]. In 2023, the new FIGO staging system incorporates comprehensive clinical, surgical, histopathological, TNM (Tumor, Lymph Node, Metastasis), prognostic high-risk factors, and molecular subtyping for staging EC [21]. This approach aims to be more closely aligned with clinical practice, providing guidance for patient surgery, adjuvant therapy, and prognosis assessment. Carolina Bezzi [36] investigated the role of machine learning (ML)-based classification using PET parameters in predicting features of EC aggressiveness, aiming at supporting the clinical decision-making process. Thus we would further our search involving the accuracy and predictive ability of PET/CT for the assessment of high-risk factors in EC patients according to 2023 new FIGO staging or use machine learning (ML) in the future.

## Conclucions

In the present study, PET/CT showed high sensitivity, specificity and accuracy in the detection of DMI, ESI and LNM, and clinicians may individualize therapy plans for endometrial cancer patients according to the cutoff values of MTV and TLG to predict risk factors in EC preoperatively. However, this study has some limitations, being retrospectively based on patients’ final histopathology reports and the statistical p-values of differences in DMI, ESI and LNM were not adjusted for multiple comparisons. Furthermore, since MRI parameters have synergic role in preoperatively predicting MI, LVSI and LNM, PET/MRI has good accuracy in preoperative staging of EC [37]. Further prospective studies with more patients and longer follow-up periods, or applying PET/MRI, are needed to investigate the potential clinical utility of the cutoff value and to verify the survival rate of patients.

### Electronic supplementary material

Below is the link to the electronic supplementary material.

Supplementary Material 1

## Data Availability

The datasets used and analysed during the current study are available in the Supplementary Information files.

## Abbreviations

^18^F-FDG: Fluorine-18 fluorodeoxyglucose
ADC: Apparent diffusion coefficient
ACRIN: American College of Radiology Imaging Network
CE: Contrast-enhanced
CT: Computed tomography
CI: confidence interval
DMI: Deep Myometrial invasion
DFS: Disease-free survival
EAH: Endometrial Atypical Hyperplasia
ESI: Endocervical stroma invasion
FIGO: The International Federation of Gynecology and Obstetrics system
FNR: False-negative rate
FP: False positive
FN: False negative
GOG: Gynecology Oncology Group
HRs: Hazard ratios
IRB: Institutional review board
LNM: Lymph node metastases
LVSI: Lymph-vascular space invasion
MRI: Magnetic resonance imaging
MTV: Metabolic tumor volume
MI: Myometrial invasion
NPV: Negative predictive value
OS: Overall survival
PALND: Paraaortic lymph node dissection
PET: Positron emission tomography
PPV: Positive predictive value
PLND: Pelvic lymph node dissection
ROC: Receiver operator characteristic curve
ROI: Region of interest
SLND: Sentinel lymph node dissection
SUV: Standardized uptake value
SUVmax: maximum standardized uptake value
SUVmean: Average of standardized uptake value
SMI: Superficial myometrial invasion
TLG: Total lesion glycolysis
TP: True positive
TN: True negative
VOI: Volume of interest

## References

[1] Siegel RL, Miller KD, Wagle NS, Jemal A, Cancer statistics. 2023. CA Cancer J Clin. 2023;73(1):17–48. 10.3322/caac.21763. 10.3322/caac.21763 36633525

[2] Connor EV, Rose PG (2018). Management strategies for recurrent endometrial Cancer. Expert Rev Anticancer Ther.

[3] Murali R, Delair DF, Bean SM, Abu-Rustum NR, Soslow RA (2018). Evolving Roles of Histologic Evaluation and Molecular/Genomic Profiling in the management of Endometrial Cancer. J Natl Compr Canc Netw.

[4] Uterine Neoplasms. The National Comprehensive Cancer Network (NCCN) clinical guidelines. Version 1.2023-December 22, 2022. 10.6004/jnccn.2023.0006 36791750

[5] Capozzi VA, Sozzi G, Rosati A, Restaino S, Gambino G, Cianciolo A, et al. Predictive score of nodal involvement in Endometrial Cancer patients: a large Multicentre Series. Ann Surg Oncol. 2021 Nov;26. 10.1245/s10434-021-11083-x. Epub ahead of print. 10.1245/s10434-021-11083-x 34837130

[6] Li W, Jiang J, Fu Y, Shen Y, Zhang C, Yao S (2021). Implications of isolated para-aortic Lymph Node Metastasis in Endometrial Cancer: a Large-Scale, Multicenter, and Retrospective Study. Front Med (Lausanne).

[7] Reijntjes B, van Suijlichem M, Woolderink JM, Bongers MY, Reesink-Peters N, Paulsen L (2022). Recurrence and survival after laparoscopy versus laparotomy without lymphadenectomy in early-stage endometrial cancer: long-term outcomes of a randomised trial. Gynecol Oncol.

[8] The Obstetrics and Gynecology Professional Committee of the Chinese Research Hospital Association (2019). Expert consensus on the treatment of early endometrial cancer with preserved fertility. Chin J Clin Obstet Gynecol.

[9] Haldorsen IS, Salvesen HB (2016). What is the best preoperative imaging for Endometrial Cancer?. Curr Oncol Rep.

[10] Stecco A, Buemi F, Cassara A, Matheoud R, Sacchetti GM, Arnulfo A (2016). Comparison of retrospective PET and MRI-DWI (PET/MRI-DWI) image fusion with PET/CT and MRI-DWI in detection of cervical and endometrial cancer lymph node metastases. Radiol Med.

[11] Yang SS, Wu YS, Chen WC, Zhang J, Xiao SM, Zhang BY (2021). Benefit of [18F]-FDG PET/CT for treatment-naive nasopharyngeal carcinoma. Eur J Nucl Med Mol Imaging.

[12] St Laurent JD, Davis MR, Feltmate CM, Goodman A, Del Carmen MG, Horowitz NE (2020). Prognostic value of preoperative imaging: comparing 18F-Fluorodeoxyglucose Positron Emission Tomography-computed tomography to computed tomography alone for Preoperative Planning in High-risk histology Endometrial Carcinoma. Am J Clin Oncol.

[13] Tsuyoshi H, Tsujikawa T, Yamada S, Okazawa H, Yoshida Y (2020). Diagnostic value of 18F-FDG PET/MRI for staging in patients with endometrial cancer. Cancer Imaging.

[14] Legros M, Margueritte F, Tardieu A, Deluche E, Mbou VB, Lacorre A, Ceuca A, Aubard Y, Monteil J, Sallee C, Gauthier T (2019). Para-aortic Lymph Node Invasion in High-risk Endometrial Cancer: performance of 18FDG PET-CT. Anticancer Res.

[15] Nakajo M, Jinguji M, Tani A, Kikuno H, Hirahara D, Togami S (2021). Application of a Machine Learning Approach for the analysis of clinical and radiomic features of pretreatment [(18)F]-FDG PET/CT to Predict Prognosis of patients with Endometrial Cancer. Mol Imaging Biol.

[16] Albano Z, Odicino, Giubbini B. Clinical and prognostic value of 18F-FDG PET/CT in recurrent endometrial carcinoma. Revista Esp De Med Nuclear e Imagen Mol. 2018.09.005. 10.1016/j.remn.2018.09.005 30573388

[17] Gee M, Atri S, Mostafa B et al. Identification of distant metastatic Disease in uterine cervical and endometrial cancers with FDG PET/CT: analysis from the ACRIN 6671/GOG 0233 Multicenter Trial. Radiol 2018 Apr;287(1):176–84. 10.1148/radiol.2017170963. 10.1148/radiol.2017170963 PMC5881639 29185901

[18] Atri M, Zhang Z, Dehdashti F, Lee SI, Gold M (2017). Utility of PET/CT to evaluate retroperitoneal lymph node Metastasis in high-risk endometrial cancer: results of ACRIN 6671/GOG 0233 trial. Radiology.

[19] Fasmer KE, Gulati A, Dybvik JA, Ytre-Hauge S, Salvesen O, Trovik J (2020). Preoperative 18F-FDG PET/CT Tumor markers outperform MRI-based markers for the prediction of lymph node metastases in primary endometrial cancer. Eur Radiol.

[20] Talhouk A, Mcconechy MK, Leung S, Li-Chang HH, Kwon JS, Melnyk N (2015). A clinically applicable molecular-based classification for endometrial cancers. Br J Cancer.

[21] Berek JS, Matias-Guiu X, Creutzberg C et al. FIGO staging of endometrial cancer: 2023 [published online ahead of print, 2023 Jun 20]. Int J Gynaecol Obstet. 2023;10.1002/ijgo.14923.

[22] Koskas M, Amant F, Mirza MR, Creutzberg CL (2021). Cancer of the corpus uteri: 2021 update. Int J Gynaecol Obstet.

[23] Rockall AG, Barwick TD, Wilson W, Singh N, Bharwani N, Sohaib A (2021). Diagnostic accuracy of FEC-PET/CT, FDG-PET/CT, and diffusion-weighted MRI in detection of nodal metastases in surgically treated endometrial and cervical carcinoma. Clin Cancer Res.

[24] Paquette M, Espinosa-Bentancourt E, Lavallee E, Phoenix S, Lapointe-Milot K, Bessette P et al. (18)F-4FMFES and (18)F-FDG PET/CT in ER + endometrial carcinomas: preliminary report. J Nucl Med 2021 Aug 19:jnumed.121.262617. 10.2967/jnumed.121.262617. Epub ahead of print. 10.2967/jnumed.121.262617 34413142

[25] Yamada S, Tsuyoshi H, Yamamoto M, Tsujikawa T, Kiyono Y, Okazawa H (2021). Prognostic value of 16alpha-(18)F-Fluoro-17beta-Estradiol PET as a predictor of Disease Outcome in Endometrial Cancer: a prospective study. J Nucl Med.

[26] Gee MS, Atri M, Bandos AI, Mannel RS, Gold MA, Lee SI (2018). Identification of distant metastatic Disease in uterine cervical and endometrial cancers with FDG PET/CT: analysis from the ACRIN 6671/GOG 0233 Multicenter Trial. Radiology.

[27] Kim HJ, Cho A, Yun M, Kim YT, Kang WJ (2016). Comparison of FDG PET/CT and MRI in lymph node staging of endometrial cancer. Ann Nucl Med.

[28] Stewart KI, Chasen B, Erwin W, Fleming N, Westin SN, Dioun S (2019). Preoperative PET/CT does not accurately detect extrauterine Disease in patients with newly diagnosed high-risk endometrial cancer: a prospective study. Cancer.

[29] Hu J, Zhang K, Yan Y, Zang Y, Wang Y, Xue F (2019). Diagnostic accuracy of preoperative (18)F-FDG PET or PET/CT in detecting pelvic and para-aortic lymph node Metastasis in patients with endometrial cancer: a systematic review and meta-analysis. Arch Gynecol Obstet.

[30] Mapelli P, Bergamini A, Fallanca F (2019). Prognostic role of FDG PET-derived parameters in preoperative staging of endometrial cancer. Función pronóstica De Los parámetros derivados de FDG PET en la estadificación preoperatoria del cáncer de endometrio. Rev Esp Med Nucl Imagen Mol (Engl Ed).

[31] Picchio M, Mangili G, Gajate AMS, Marzi PD, Messa C (2010). High-grade endometrial cancer: value of [(18)F]FDG PET/CT in preoperative staging. Nucl Med Commun.

[32] Erdogan M, Erdemoglu E, Evrimler Ş, Hanedan C, Şengül SS (2019). Prognostic value of metabolic Tumor volume and total lesion glycolysis assessed by 18F-FDG PET/CT in endometrial cancer. Nucl Med Commun.

[33] Shim SH, Kim DY, Lee DY, Lee SW, Park JY, Lee JJ (2014). Metabolic tumour volume and total lesion glycolysis, measured using preoperative18F-FDG PET/CT, predict the recurrence of endometrial cancer. BJOG: An International Journal of Obstetrics & Gynaecology.

[34] Concin N, Matias-Guiu X, Vergote I, Cibula D, Mirza MR, Marnitz S (2021). ESGO/ESTRO/ESP guidelines for the management of patients with endometrial carcinoma. Int J Gynecol Cancer.

[35] León-Castillo A, Britton H, McConechy MK (2020). Interpretation of somatic POLE mutations in endometrial carcinoma. J Pathol.

[36] Bezzi C, Bergamini A, Mathoux G (2023). Role of machine learning (ML)-Based classification using conventional ^18^F-FDG PET parameters in Predicting Postsurgical features of Endometrial Cancer aggressiveness. Cancers (Basel).

[37] Ironi G, Mapelli P, Bergamini A, Fallanca F, Candotti G, Gnasso C (2022). Hybrid PET/MRI in staging Endometrial Cancer: Diagnostic and Predictive Value in a prospective cohort. Clin Nucl Med.

