# Supplementary material for: Retrospective analysis of the 18F-FDG PET/CT cutoff value for metabolic parameters was performed as a prediction model to evaluate risk factors for endometrial cancer
Source: Radiat Oncol. 2023 Dec 4;18:196. doi: 10.1186/s13014-023-02382-6 (PMC10696876; doi:10.1186/s13014-023-02382-6)
Supplement: Supplementary file 1 — Supplementary Material 1 [file 13014_2023_2382_MOESM1_ESM.docx]

**Supplementary Table 1**

2009 and 2023 International Federation of Gynecology and Obstetrics Surgical Staging Systems for Endometrial Cancer.

| 2023 Stage | Description | 2009 Stage | Description |
| --- | --- | --- | --- |
| I | Confined to the uterine corpus and ovary | I | Tumor conﬁned to the corpus uteri, including endocervical glandular involvement |
| IA | Disease limited to the endometrium OR non-aggressive histological type, i.e. low-grade endometroid, with invasion of less than half of myometrium with no or focal lymphovascular space involvement (LVSI) OR good prognosis disease | IA | Tumor limited to the endometrium or invading less than half the myometrium |
| IA1 | Non-aggressive histological type limited to an endometrial polyp OR confined to the endometrium |  |  |
| IA2 | Non-aggressive histological types involving less than half of the myometrium with no or focal LVSI |  |  |
| IA3 | Low-grade endometrioid carcinomas limited to the uterus and ovary |  |  |
| IB | Non-aggressive histological types with invasion of half or more of the myometrium, and with no or focal LVSI | IB | Tumor invading one half or more of the myometrium |
| IC | Aggressive histological types limited to a polyp or confined to the endometrium |  |  |
| II | Invasion of cervical stroma with extrauterine extension OR with substantial LVSI OR aggressive histological types with myometrial invasion | II | Tumor invading the stromal connective tissue of the cervix but not extending beyond the uterus. Does NOT include endocervical glandular involvement |
| IIA | Invasion of the cervical stroma of non-aggressive histological types |  |  |
| IIB | Substantial LVSI of non-aggressive histological types |  |  |
| IIC | Aggressive histological types with any myometrial involvement |  |  |
| III | Local and/or regional spread of the tumor of any histological subtype | III | Tumor involving serosa, adnexa, vagina, or parametrium |
| IIIA | Invasion of uterine serosa, adnexa, or both by direct extension or metastasis | IIIA | Tumor involving the serosa and/or adnexa (direct extension or metastasis) |
| IIIA1 | Spread to ovary or fallopian tube (except when meeting stage IA3 criteria) |  |  |
| IIIA2 | Involvement of uterine subserosa or spread through the uterine serosa |  |  |
| IIIB | Metastasis or direct spread to the vagina and/or to the parametria or pelvic peritoneum | IIIB | Vaginal involvement (direct extension or metastasis) or parametrial involvement |
| IIIB1 | Metastasis or direct spread to the vagina and/or the parametria |  |  |
| IIIB2 | Metastasis to the pelvic peritoneum |  |  |
| IIIC | Metastasis to the pelvic or para-aortic lymph nodes or both | IIIC |  |
| IIIC1 | Metastasis to the pelvic lymph nodes | IIIC1 | Regional lymph node metastasis to pelvic lymph nodes |
| IIIC1i | Micrometastasis |  |  |
| IIICii | Macrometastasis |  |  |
| IIIC2 | Metastasis to para-aortic lymph nodes up to the renal vessels, with or without metastasis to the pelvic lymph nodes | IIIC2 | Regional lymph node metastasis to para-aortic lymph nodes, with or without positive pelvic lymph nodes |
| IIIC2i | Micrometastasis |  |  |
| IIIC2ii | Macrometastasis |  |  |
| IV | Spread to the bladder mucosa and/or intestinal mucosa and/or distance metastasis | IV |  |
| IVA | Invasion of the bladder mucosa and/or the intestinal/bowel mucosa | IVA | Tumor invading the bladder mucosa and/or bowel mucosa |
| IVB | Abdominal peritoneal metastasis beyond the pelvis | IVB | Distant metastasis (includes metastasis to inguinal lymph nodes, intraperitoneal disease, lung, liver, or bone). (It excludes metastasis to pelvic or para-aortic lymph nodes, vagina, uterine serosa, or adnexa). |
| IVC | Distant metastasis, including metastasis to any extra-or intra-abdominal lymph nodes above the renal vessels, lungs, liver, brain, or bone |  |  |

Supplementary Table 2 Disease progression in EC patients analyzed with MTV of MI and LNM.

|  |  | Histologic | | Follow-up | Progression | Survial | Lesion | MI | | | | LNM | | | | |
| --- | --- | --- | --- | --- | --- | --- | --- | --- | --- | --- | --- | --- | --- | --- | --- | --- |
| Age | FIGO | Subtype | Grade | Follow-up | Progression |  |  | SUVmax | SUVmean | MTV(ml) | TLG(g) | SUVmax | SUVmean | MTV(ml) | TLG(g) |  |
| 77 | IVA | E | G3 | 45 | 36 | 0 | bladder mucosa | 12.47 | 6.52 | 43.48 | 283.4896 | 8.19 | 3.89 | 30.51 | 118.6839 |  |
| 54 | IIIC2 | E | G3 | 49 | 44 | 0 | para-aortic lymph nodes | 10.06 | 6.19 | 44.81 | 277.3739 | 10.01 | 4.68 | 46.13 | 215.8884 |  |
| 70 | IVB | NE | G3 | 31 | 19 | 1 | lung | 12.64 | 5.77 | 40.29 | 232.4733 | 6.6 | 5.12 | 27.34 | 139.9808 |  |
| 67 | IVA | E | G3 | 45 | 43 | 0 | bladder mucosa | 13.98 | 5.77 | 40.88 | 235.8776 | 9.15 | 6.29 | 35.91 | 225.8739 |  |
| 83 | IVA | E | G3 | 43 | 40 | 1 | bowel mucosa | 15.93 | 4.37 | 44.46 | 194.2902 | 10.41 | 5.39 | 26.71 | 143.9669 |  |
| 52 | IVB | NE | G3 | 39 | 34 | 1 | bladder mucosa | 15.17 | 6.65 | 34.72 | 230.888 | 7.78 | 4.57 | 35.71 | 163.1947 |  |
| 78 | IIIC2 | E | G3 | 45 | 33 | 1 | lung | 10.1 | 5.88 | 31.09 | 182.8092 | 12.59 | 6.74 | 31.37 | 211.4338 |  |
| 62 | IVA | E | G3 | 46 | 39 | 0 | bladder mucosa | 8.25 | 4.72 | 39.97 | 188.6584 | 12.54 | 4.17 | 25.6 | 106.752 |  |
| 70 | IVB | NE | G3 | 34 | 17 | 1 | Lung,  bone | 9.35 | 4.5 | 34.94 | 157.23 | 9.24 | 3.2 | 27.4 | 87.68 |  |
| 74 | IVB | NE | G3 | 23 | 14 | 1 | bone | 12 | 3.51 | 26.06 | 91.4706 | 7.38 | 4.19 | 40.35 | 169.0665 |  |
| 72 | IVA | NE | G3 | 42 | 33 | 0 | bowel mucosa | 6.77 | 5.01 | 31.24 | 156.5124 | 11.36 | 6.74 | 42.49 | 286.3826 |  |
| 77 | IVA | E | G3 | 39 | 38 | 0 | bladder, bowel mucosa | 6.77 | 5.01 | 31.24 | 156.5124 | 11.36 | 6.74 | 42.49 | 286.3826 |  |
| 70 | IIIC2 | NE | G3 | 45 | 31 | 0 | lung | 8.77 | 2.96 | 35.42 | 104.8432 | 13.11 | 6.33 | 33.3 | 210.789 |  |
| 38 | IIIC1 | E | G2 | 45 | 41 | 0 | lung | 13.23 | 4.66 | 30.28 | 141.1048 | 7.56 | 3.9 | 30.49 | 118.911 |  |
| 66 | IIIA | E | G3 | 51 | 44 | 0 | lung | 10.64 | 4.87 | 34.1 | 166.067 | 11.21 | 4.86 | 35.03 | 170.2458 |  |
| 50 | II | NE | G3 | 47 | 45 | 0 | bone | 12.47 | 4.66 | 27.74 | 129.2684 | 10.1 | 4.23 | 36.57 | 154.6911 |  |
| 78 | IIIC1 | E | G2 | 41 | 37 | 0 | para-aortic lymph nodes | 13.8 | 4.31 | 32.99 | 142.1869 | 2.2 | 1.91 | 17.08 | 32.6228 |  |
| 75 | II | E | G1 | 47 | 26 | 1 | pelvic Lymph nodes | 12.1 | 6.59 | 42.55 | 280.4045 | 1.51 | 1.28 | 9.06 | 11.5968 |  |
| 62 | IIIC2 | NE | G3 | 47 | 41 | 0 | lung | 13.19 | 6.46 | 33.25 | 214.795 | 1.1 | 1.4 | 17.64 | 24.696 |  |
| 66 | IIIA | E | G3 | 45 | 42 | 0 | pelvic Lymph nodes | 11.24 | 4.35 | 26.5 | 115.275 | 1.89 | 1.52 | 13.6 | 20.672 |  |
| 55 | IIIC | E | G2 | 42 | 43 | 0 | para-aortic lymph nodes | 0.72 | 0.73 | 10.69 | 7.8037 | 1.15 | 2.16 | 10.98 | 23.7168 |  |
| 51 | IIIB | E | G | 50 | 41 | 0 | Liver | 1.9 | 0.5 | 15.51 | 7.755 | 1.96 | 2.05 | 16 | 32.8 |  |
| 70 | IIIB | E | G3 | 44 | 29 | 1 | Lung | 1.48 | 2.34 | 17.07 | 39.9438 | 1.24 | 1.28 | 12.3 | 15.744 |  |
| 85 | IIIC1 | E | G3 | 41 | 45 | 1 | myocardial infarction | 9.06 | 4.35 | 33.27 | 144.7245 | 12.3 | 4.3 | 26.23 | 112.789 |  |
| 80 | IIIC2 | NE | G3 | 47 | 50 | 1 | myocardial infarction | 8.4 | 4.47 | 28.11 | 125.6517 | 9.71 | 5.07 | 46.51 | 235.8057 |  |
| 85 | IA | E | G2 | 45 | 45 | 1 | myocardial infarction | 12.5 | 3.51 | 32.65 | 114.6015 | 9.45 | 4.98 | 44.64 | 222.3072 |  |
| 80 | IB | NE | G2 | 43 | 43 | 1 | myocardial infarction | 10.29 | 5.17 | 38.52 | 199.1484 | 1.45 | 1.47 | 14.74 | 21.6678 |  |
| 87 | II | E | G2 | 42 | 42 | 1 | pulmonary embolism | 11.31 | 3.02 | 36.12 | 109.0824 | 1.9 | 1.35 | 10.59 | 14.2965 |  |
| 80 | IIIA | E | G1 | 47 | 47 | 1 | myocardial infarction | 1.85 | 1.59 | 22.16 | 35.2344 | 2.09 | 2.24 | 20.63 | 46.2112 |  |
| 86 | IA | E | G1 | 43 | 43 | 1 | pulmonary embolism | 2.21 | 1.68 | 22.68 | 38.1024 | 2.18 | 0.88 | 9.36 | 8.2368 |  |

E: NO-endometrioid

NE: NO-endometrioid
